# Is objectively measured light-intensity physical activity associated with health outcomes after adjustment for moderate-to-vigorous physical activity in adults? A systematic review

**DOI:** 10.1186/s12966-018-0695-z

**Published:** 2018-07-09

**Authors:** Shiho Amagasa, Masaki Machida, Noritoshi Fukushima, Hiroyuki Kikuchi, Tomoko Takamiya, Yuko Odagiri, Shigeru Inoue

**Affiliations:** Department of Preventive Medicine and Public Health, Tokyo Medical University, Tokyo, Japan

**Keywords:** Accerelometry, Epidemiology, Public health, Lifestyle activity, Physical activity

## Abstract

**Background:**

An increasing number of studies have demonstrated that light-intensity physical activity (LPA) confers health benefits after adjustment for moderate-to-vigorous physical activity (MVPA). The purpose of this systematic review was to summarize existing epidemiological evidence on associations of objectively measured LPA with health outcomes in adults.

**Methods:**

This review was conducted in accordance with the Preferred Reporting Items for Systematic Reviews and Meta-Analyses guidelines. We searched on PubMed, Web of Science, CINAL, and Cochrane Library for articles analyzing the association between objectively determined LPA and health outcomes that were published up to January 2017. Data were extracted regarding authors, publication year, country of survey, study setting, number of participants, study design, physical activity (PA) assessment (type of accelerometer and intensity), health outcomes, confounders, and results (summary measures and association). A coding system was used to summarize the results.

**Results:**

Of the 3254 studies identified, 24 cross-sectional and 6 longitudinal studies were included in this review. Most of the studies targeted the Western population. LPA was inversely associated with all-cause mortality risk and associated favorably with some cardiometabolic risk factors including waist circumference, triglyceride levels, insulin, and presence of metabolic syndrome. Only a small amount of data were available on mental health and cognitive function.

**Conclusions:**

LPA appears to be beneficially associated with important health outcomes after adjustment for MVPA in the adult population. Although current global PA guidelines recommend only MVPA, promoting LPA may confer additional health benefits.

**Electronic supplementary material:**

The online version of this article (10.1186/s12966-018-0695-z) contains supplementary material, which is available to authorized users.

## Background

It is well documented that moderate-to-vigorous physical activity (MVPA) is effective in the prevention of major non-communicable diseases including type 2 diabetes, coronary heart disease, stroke, and some types of cancer [1]. Current global physical activity (PA) guidelines recommend that adults engage in at least 150 min of MVPA in a week or 75 min of vigorous PA in a week in bouts of at least 10 min to achieve health benefits [2]. Even though LPA has great potential for increasing total PA levels (i.e., PA energy expenditure) [3, 4], the effect of light-intensity PA (LPA) has remained controversial. It has been reported that people spend a significant portion of their time in sedentary behavior (SB) and LPA and only a little time in MVPA [5–7]. According to the National Health and Nutrition Examination Survey (NHANES) data, within a day (24 h), adults spent an average of 7.7 h in SB, 7.8 h in LPA, 0.2 h in MVPA, and 8.3 h in sleep [6]. Thus, the clarification of the effects of LPA is crucial to promote public health.

The topic of epidemiological studies has shifted from MVPA to the health benefits of LPA, owing to the development of accelerometry techniques in epidemiological studies [8, 9]. Even though several studies have confirmed the potential health benefits of self-rated LPA (e.g., housework) [10, 11], recalling the time spent in LPA dispersed throughout the day may be difficult compared with MVPA lasting for at least 10 min. Objective assessment can record more detailed and accurate patterns of personal daily activity [9, 12]. Emerging evidence suggests that objectively determined LPA is associated with all-cause mortality [13], cardiometabolic biomarkers [14], and plasma glucose levels [15] after adjustment for MVPA time.

To date, there have been no comprehensive reviews published to our knowledge on whether objectively measured LPA is associated with health outcomes after adjustment for MVPA. Fuzeki et al. [16] recently reviewed the health benefits of objectively measured LPA, but they focused on only the NHANES dataset and hence generalizability of the data is limited. Moreover, most studies, including the review by Fuzeki et al. did not adjust for MVPA, which may confound the associations of LPA with health. Therefore, the aim of this present review was to systematically examine associations of objectively assessed LPA and health outcomes after adjustment for MVPA in adults.

## Methods

### Information sources and searches

Information searches were performed in accordance with the Preferred Reporting Items for Systematic Reviews and Meta-Analyses (PRISMA) guidelines [17]. Studies were obtained through searching the following four electronic databases: PubMed, Web of Science, CINAL, and Cochrane Library. We performed the search on February 2, 2017, using the search terms presented in Additional file 1. These search terms are created based on a previous study [18]. A hand search was also carried out to supplement the electronic database searches.

### Study selection

Inclusion and exclusion criteria were determined prior to undertaking the review. We included the following observational studies: 1) those that assessed associations of objectively measured LPA with at least 1 health outcomes, 2) targeted adults, and 3) were written in English or Japanese. We included studies that analyzed the effects of various intensities of PA, provided that they also analyzed the association between LPA and the outcomes. No limitation on publication year was included.

Studies were excluded if they met the following criteria: 1) targeted only a particular populations in the medical setting (e.g., patients, survivors, or pregnant women), 2) investigated the association with physical fitness, 3) did not control for covariates, and 4) was not an original research article.

### Data extraction and study quality assessment

Data extraction and study quality assessment was performed by two independent researchers (SA and MM), and differences in judgement between the two researchers were discussed until they reached a consensus. The extracted data included the following information: author(s), publication year, county of survey, population (sex and age), number of participants, study design, PA assessment (intensity, type of accelerometer, LPA cut off points, and duration of LPA), health outcomes, and results (summary measures and association).

The quality of the included studies was assessed using the Quality Assessment Tool used in a previous review [16]. Briefly, this assessment tool includes a 12-item checklist, and items were coded as ‘present (1)’ or ‘absent/ unclear (0)’. Studies scoring 10 points and above, 9–6 points, and below 6 points were classified as high, moderate, and low quality, respectively. Because of the heterogeneity of the study designs, outcome variables, statistical analyses, and the context in which the data were collected, a meta-analysis was not applied. Therefore, a narrative synthesis of the evidence was conducted. The data synthesis included findings from the studies analyzed, such as study design, sample, accelerometer (definition of LPA), exposure, outcomes (direction), confounders, and quality assessment.

A coding system created by Sallis et al. [19] was used to summarize the association between LPA and health outcomes. The results were classified as follows: “no association (0)” if 0%–33% of the papers reported a statistically significant difference between LPA and outcomes, “indeterminate (?)” if 34%–59% of the papers indicated a statistically significant difference, and “favorable association (+)/unfavorable association (-)” if 60–100% of the papers showed a statistically significant favorable/ unfavorable difference. When more than four studies supported an association or no association, it was coded as “00”, “++”, or “−−”. The “??” was used if there were inconsistent findings. We used the results of the final model of multivariate analyses (fully adjusted model) if multiple models were run in one study. Studies analyzing multiple health benefits were included in each of the relevant items.

## Results

### Search and selection

**Fig. 1:**
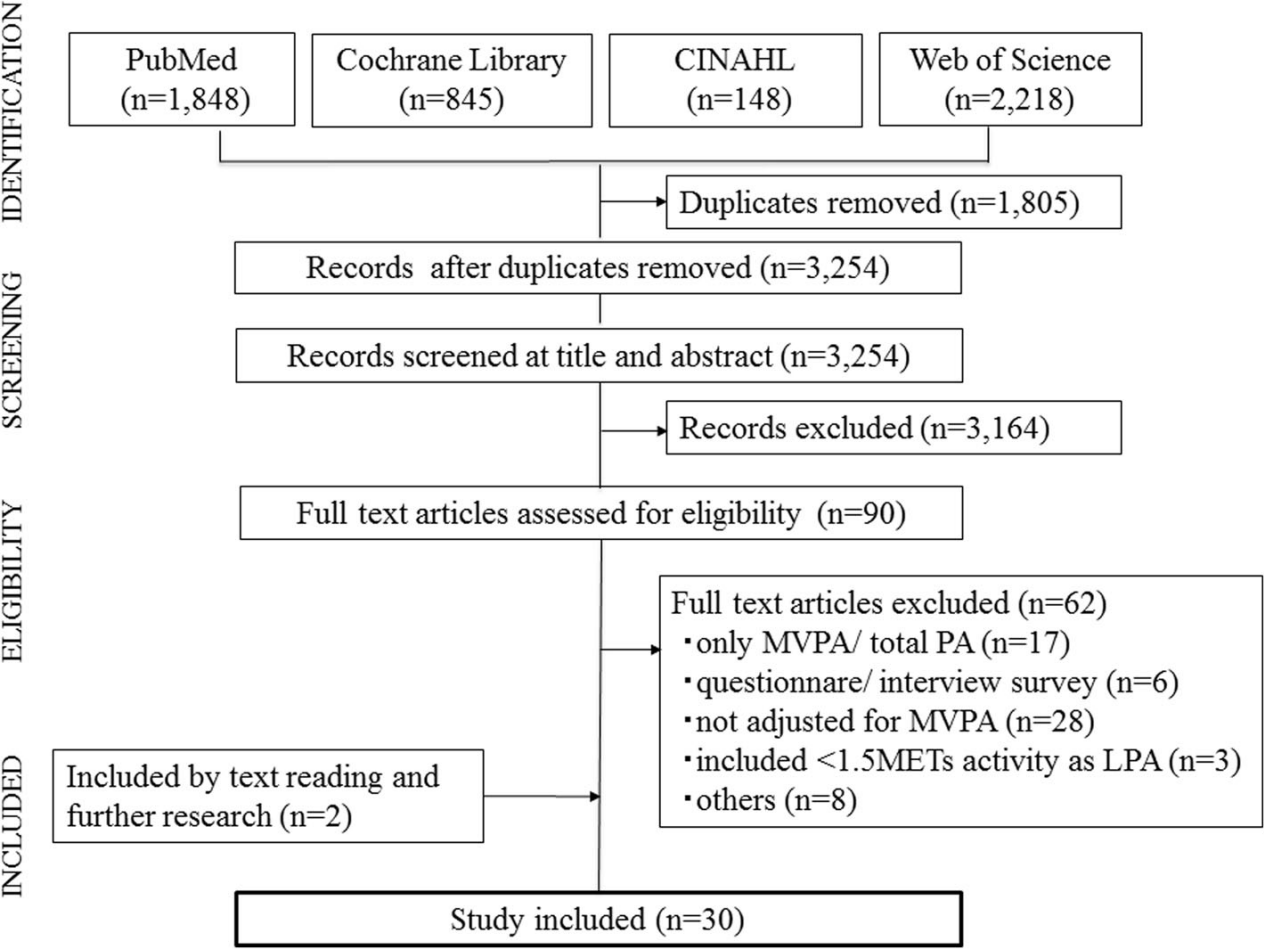
Flow diagram of the inclusion of articles in this study. PA: physical activity, LPA: light-intensity physical activity, MVPA: moderate-to-vigorous physical activity

A flow diagram of article inclusion is shown in Fig. 1. A total of 5059 potential studies were identified through electronic database searching (1848 from PubMed, 2218 from Web of Science, 845 from Cochrane Library, and 148 from CINAL). After removing duplicate records, 3254 articles remained. Of these, 3164 articles were excluded by means of title and abstract screening and 90 full text articles were assessed for eligibility. After reading the full text, 28 were concluded to meet the inclusion criteria, and two were added by further searching. As a result, 30 articles were included in this review.

### Study characteristics and quality assessment

The characteristics of the studies reviewed are described in Table 1. Among the 30 eligible studies, 17 included adults (> 18 years) (men: 2; women: 2; and both: 13), four included older adults (men: 1; women: 0; and both: 3), and nine included both adults and older adults (men: 0; women: 1; and both: 8). We were unable to summarize the results by age group (i.e., adults or older adults) owing to limited data. The eligible papers were published between 2007 and February 2017, and most of them (*n* = 24, 80%) were cross-sectional studies. Seventeen of the included studies were from the United States, and the rest were from the United Kingdom (*n* = 3), Australia (n = 3), Canada (*n* = 1), Belgium (*n* = 1), Finland (*n* = 1), Japan (*n* = 1), Taiwan (*n* = 1), Sweden (*n* = 1), and Saudi Arabia (*n* = 1). Study samples ranged from 50 to 5562 and half of the studies (*n* = 15) had a sample of more than 1000 participants. Most of the studies were of moderate quality.

**Table 1:** Summary of all studies included in this systematic review

Author Year	Study design	Participants	Accelerometer (definition of LPA)	Exposure(s)	Outcome measurements (direction)^a^	Confounders	Study quality
Adults							
Loprinzi PD. 2016 [26]	Cross- sectional (NHANES 2005–2006)	2048 adults in the U.S. (≥ 20 years)	ActiGraph 7164 (100 to 2019 CPM)	Every 60-min increase/day in LPA	Multimorbidity (+): OR 0.87 (0.79, 0.96)	Age, sex, race-ethnicity, poverty-to-income ratio, MVPA, eating a healthy diet, smoking, and meeting sleep guidelines	Moderate
Robson J., et al. 2015 [27]	Cross-sectional (NHANES 2003–2004 and 2005–2006)	1974 adults in the U.S. (≥ 20 years)	Actigraph AM-7164 (100 to 2019 CPM)	Every 30-min increase/day in bouted LPA and sporadic LPA	Bouted LPA metabolic syndrome (+): OR 0.96 (0.93, 0.99), WC (+): OR 0.96 (0.93, 0.98), triglycerides (+):OR 0.93 (0.90, 0.96), HDL(0): OR 0.98 (0.94, 1.01), BP (0): OR 0.98 (0.94, 1.02), and glucose (0): OR 0.97 (0.94, 1.01) Sporadic LPA metabolic syndrome (0): OR 0.91 (0.81, 1.03), WC (0): OR 0.88 (0.77, 1.00), triglycerides (0):OR 0.94 (0.83, 1.06), HDL(0): OR 0.99 (0.88, 1.11), BP (0): OR 0.96 (0.80, 1.12), and glucose (0): OR 0.99 (0.86, 1.14)	Bouted LPA age, sex, ethnicity, poverty–income ratio, alcohol, smoking, and the other physical activity variables with the exception of embedded MVPA Sporadic LPA age, sex, ethnicity, poverty–income ratio, alcohol, smoking, and the other physical activity variables with the exception of bouted MVPA	High
Healy GN., et al. 2007 [15]	Cross-sectional (AusDiab study 2004–2005)	173 Australian adults (mean 53.3 years)	Actigraph (100 to 1951 CPM)	LPA (hours/day)	2-h plasma glucose (+): β −0.22 (− 0.42, − 0.03), adjusted R^2^ 0.17	Age, sex, height, WC, time accelerometer worn, accelerometer unit, family history of diabetes, alcohol intake, education, income, smoking status, and MVPA	Moderate
Hawkins MS., et al. 2013 [20]	Cross-sectional (NHANES 2003–2004)	463 adults in the U.S. with normal ankle–brachial index (56.0 ± 0.5 years)	ActiGraph AML-7164 (100 to 1951 CPM)	LPA (min/day)	Framingham risk score (0): β 0.0016	Age, sex, race, lipid-lowering medication, BMI, diabetes status, smoking status, CRP, lipids, SB and MVPA	Moderate
Riou MÈ., et al. 2014 [44]	Cohort (part of the MONET study)	65 Canadian women (47–54 years)	Actical	Tertile of LPA (min/week)	Body weight (0), BMI (0),WC (0), fat mass (+), fat-free mass (0), percent body fat (+), peripheral fat mass (0), and central fat mass (+)	MVPA and total energy intake	Moderate
Green AN., et al. 2014 [31]	Cross-sectional	50 young women in the U.S. (24.0 ± 4.8 years)	ActiGraph GT3X+ (150 to 2689 CPM)	LPA (min/day)	Triglycerides (+), lipid accumulation product (+), and HOMA R (0)	MVPA, VO_2_peak, and body mass	Moderate
Scheers T., et al. 2013 [42]	Cross-sectional	370 Flemish adults (41.7 ± 9.8 years)	SenseWear Pro 3 Armband	LPA (hours/day)	Metabolic syndrome (0): OR 0.88 (0.62, 1.26), abdominal obesity (0): OR 0.89 (0.67, 1.18), hypertriglyceridemia (0): OR 0.85 (0.64, 1.13), HDL-C (0): OR 1.12 (0.82, 1.51), hypertension (0): OR 0.93 (0.75, 1.15), and hyperglycemia (0): OR 1.15 (0.78, 1.70)	Sex, age, education, smoking status, alcohol consumption, and MVPA	Moderate
Salonen MK., et al. 2015 [43]	Cross-sectional (AYLS)	737 Finnish young adults (24.4 ± 0.6 years)	SenseWear Armband	LPA	Metabolic syndrome (0): OR 1.26 (0.96, 1.66)	Age, sex, smoking, MVPA, and education	Moderate
Loprinzi PD., et al. 2013 [28]	Cross-sectional (NHANES 2003–2004, 2005–2006)	1672 men in the U.S. (mean 55.6 years)	ActiGraph 7164 (100 to 2019 CPM)	Every 60-min increase/day in ≥1-min of LPA and ≥ 10-min of LPA	≥1-min of LPA Serum prostate-specific antigen concentrations (+): OR 0.82 (0.68, 1.00) ≥10-min of LPA Serum prostate-specific antigen concentrations (0): OR 0.86 (0.73, 1.02)	Age, BMI, race, education, marital status, and dietary, biological, immunological variables, and MVPA	High
Alkahtani S., et al. 2015 [21]	Cross-sectional	84 Saudi men (37.6 ± 8.8 years)	ActiGraph wGT3X-BT (100 to 1951 CPM)	LPA	HDL-C (+): β −4.7	10-min MVPA	Moderate
Matthews CE., et al. 2016 [36]	Cohort (NHANES 2003–2006)	4840 adults in the U.S. (56.8 ± 0.4 years)	Actigraph 7164 (100 to 759 CPM)	Replace 1 h of SB with LPA LPA (3, 4, 5, ≥6 h/day) (ref. 3 h/day)	Mortality (+): HR 0.82 (0.73, 0.92) Mortality (+): HR 0.79 (0.7, 0.9) in 4 h/day, HR 0.77 (0.6, 1.0) in 5 h/day, HR 0.89 (0.6, 1.3) in ≥6 h/day	Age, race, education, sex, smoking, alcohol, diabetes, coronary artery disease, cancer, stroke, mobility limitations, BMI, and MVPA	High
Camhi SM., et al. 2011 [32]	Cross-sectional (NHANES 2005–2006)	1371 adults in the U.S. (47.1 ± 0.9 years)	ActiGraph AM-7164 (760 to 2019 CPM)	Every 30 min/day increase in LPA	Triglycerides (+): β 0.89 (0.83, 0.97), HDL-C (+): β 0.87 (0.83, 0.92), SBP (0): β 0.95 (0.85, 1.06), glucose (0): β 0.93 (0.85, 1.03), WC (+): β 0.88 (0.83, 0.94), metabolic syndrome (+): β 0.87 (0.80, 0.96), hypertension (0): β 0.90 (0.77, 1.04), and diabetes (+): β 0.67 (0.55, 0.82)	Age, ethnicity, sex and MVPA recommendations (< 30 and ≥ 30 MVPA min/day)	Moderate
Loprinzi PD., et al. 2014 [29]	Cross-sectional (NHANES 2003–2006)	1703 adults in the U.S. (mean 60.6 years)	Actigraph 7164 (100 to 2019 CPM)	LPA	BMI (+): β-0.01 (− 0.01, − 0.004), WC (+): β − 0.01 (− 0.02, − 0.009), CRP (0): β 0.00003 (− 0.001, 0.001), white blood cell (0): β − 0.0005 (− 0.002, 0.001), triglycerides (0): β − 0.07 (− 0.18, 0.03), HDL-C (0): β 0.001 (− 0.01, 0.01), glucose (0): β − 0.02 (− 0.05, 0.01), homocysteine (0): β − 0.004 (− 0.008, − 0.00001), and HbA1_C_ (0): β − 0.0002 (− 0.0007, 0.0001)	Age, race-ethnicity, sex, BMI, cotinine, comorbidity index, poverty-to-income ratio, MVPA, and accelerometer wear time	Moderate
Chastin SFM., et al. 2015 [24]	Cross-sectional (NHANES 2005–2006)	1937 adults in the U.S. (21–64 years)	Actigraph 7164 (100 to 1951 CPM)	LPA	BMI (−): γ 0.98, WC (0): γ 0.96, triglycerides (+): γ − 0.21, insulin (+): γ − 0.13, HOMA R (+): γ − 0.15, SBP (0): γ 1.30, DBP (0): γ 0.70, HDL-C (0): γ − 0.01, LDL-C (0): γ − 0.10, CRP (0): γ-0.06, and glucose (0): γ − 0.01	Age, sex, ethnicity/race, self-reported health, diagnosis of health conditions, educational level, socio economic status, smoking status, alcohol consumption, total daily average dietary calorie intake, fat intake, caffeine intake, usage of medications for high blood pressure or diabetes, and time spent in other behaviors	Moderate
Nelson RK., et al. 2013 [39]	Cross-sectional (NHANES 2003–2004)	402 adults in the U.S. (32.6 ± 0.6 years)	ActiGraph AM-7164 (260 to 1952 CPM)	LPA	HOMA R (0): β − 0.0154	Adiposity, sex, age, and MVPA	Moderate
Ekblom-Bak E., et al. 2016 [40]	Cross-sectional (Swedish CArdioPulmonary bioImage study)	836 Swedish adults (50–64 years)	Actigraph GT3X+ (200 to 2689 CPM)	Replace 10-min of SB with LPA	Metabolic syndrome (+): β 0.96 (0.93, 0.98), WC (+): β 0.97 (0.95, 0.98), triglyceride (+): β 0.97 (0.94, 0.99), HDL-C (+): β 0.95, 0.92, 0.98), BP (0): β 1.00 (0.97, 1.02), and glucose (0): β 0.99 (0.97, 1.01)	Sex, age, educational level, smoking habits, perceived psychological stress, energy intake, wear time, MPA, and VPA	Moderate
Kim J., et al. 2013 [45]	Cross-sectional	483 Japanese adults (30–64 years)	Active Style Pro (1.6–2.9 METs)	LPA (hours/day) or tertile of LPA (< 11.1, 11.2–14.5, ≥ 14.6 METs-h day)(ref. < 11.1 METs-h day)	Metabolic syndrome (+): β − 0.249 (− 0.448, − 0.051), WC (+): β − 0.827 (− 1.518, − 0.137), SBP (0): β − 3.035 (− 6.695, 0.625), DBP (0): β − 0.215 (− 0.956, 0.525), glucose (0): β − 0.790 (− 1.993, 0.413), triglyceride (0): β − 3.582 (− 8.424, 1.259), HDL-C (+): β 1.118 (0.188, 2.049), dyslipidemia (+): OR 0.68 (0.39, 1.17), 0.39 (0.20, 0.74), hypertension (0) OR 0.98 (0.61, 1.58), 0.97 (0.59, 1.60) and abdominal obesity (+): OR 0.46 (0.28, 0.76), 0.50 (0.30, 0.84).	Age, sex, smoking status, calorie intake, accelerometer wear time and MVPA	Moderate
Older adults							
Johnson LG., et al. 2016 [33]	Cross-sectional (Tasmanian Older Adult Cohort Study)	188 community-dwelling older adults in Australia (64.0 ± 7.3 years)	ActiGraph GT1M (251 to 1951 CPM)	LPA (min/day)	Cognitive performance (+): β − 0.114 (− 0.198, − 0.030)	Age, sex, smoking history, alcohol intake, educational achievement, MVPA, and neuropsychological functioning	Moderate
Ku PW., et al. 2016 [22]	Cohort	307 community-dwelling older adults in Taiwan (≥ 65 years)	Actigraph GT3X+ (100 to 1951 CPM)	LPA	Dimensions of well-being: psychological (+), learning and growth (+), and social (+), general (0), physical (0), independence (0), material (0), and environmental (0)	Age, sex, education, living status, BMI, number of chronic disease, general or specific dimension of well-being, MVPA, and accelerometer wear time	Moderate
Jefferis BJ., et al. 2016 [35]	Cross-sectional (British Regional Heart Study)	1009 older men in the U.K. (78.5 ± 4.7 years)	Actigraph GT3X (100 to 1040 CPM)	LPA (min/day) bouts in 1–9 min and ≥ 10 min	1–9 min bouts BMI (+): β − 0.012 (− 0.019, − 0.006), WC (+): β − 0.025 (− 0.044, − 0.006), fat mass index (+): β − 0.007 (− 0.013, − 0.002), insulin (+): β − 0.017 (− 0.028, − 0.006), and metabolic syndrome (0): β 0.998 (0.993, 1.003) ≥10 min bouts BMI (0): β 0.007 (− 0.008, 0.021), WC (0): β − 0.010 (− 0.052, 0.033), fat mass index (0): β − 0.003 (− 0.015, 0.009), insulin (−): β 0.028 (0.004, 0.052), and metabolic syndrome (0): β 1.002 (0.990, 1.013)	Age, social class, living alone, smoking, alcohol use, region of residence, season of accelerometer wear, MVPA, and accelerometer wear time	High
Foong et al. 2014 [34]	Cross-sectional (Tasmanian Older Adult Cohort Study)	636 Australian older adults (66.3 ± 7.1 years)	ActiGraph GT1M (251 to 1951 CPM)	Every 10-min increase in LPA	Body fat (+): β − 169 (− 277, − 61), trunk fat (+): β − 104 (− 169, − 39), and BMI (0): β − 0.04 (− 0.10, 0.02)	Age, sex, SB, MPA, and VPA.	Moderate
Adults and older adults							
Hamer M., et al. 2014 [37]	Cross-sectional (Whitehall II epidemiological cohort)	445 adults in the U.K. (66 ± 6 years)	ActiGraph GT3X (200 to 1998 CPM)	Replace 10-min of SB with LPA	Glycated hemoglobin (0): β 0.001 (0.006, − 0.009), BMI (0): β − 0.002 (− 0.059, 0.056), HDL-C (0): β 0.005 (− 0.001, 0.010), and triglyceride (0): β − 0.004 (− 0.014, 0.006)	Age, sex, smoking, employment grade, MVPA, total wear time, and current statin use	Moderate
Fishman EI., et al. 2016 [30]	Cohort (NHANES 2003–2006& National Death Index)	3029 U.S adults (50–79 years)	ActiGraph AM-7164 (100 to 2019 CPM)	Replace 10-min of SB with LPA Replace 30-min of SB with LPA	Mortality (+) HR: 0.92 (0.89, 0.94) Mortality (+) HR: 0.80 (0.75, 0.85)	Total minutes of device wear time, minutes of MVPA, baseline age, sex, race/ethnicity, education, smoking, BMI, mobility limitations, and prevalent chronic disease	Moderate
Lynch BM., et al. 2011 [23]	Cross-sectional (NHANES 2003–2006)	1024 women in the U.S. (63.0 ± 9.4 years)	Actigraph 7164 (100 to 1951 CPM)	LPA (hours/day: < 4.48, 4.48 to 5.49, 5.49 to < 6.48, ≥6.48)	BMI (+), WC (+), CRP (0), glucose (0), insulin (0), and HOMA R (0)	Age, MVPA, and BMI: ethnicity, alcohol intake, age at first birth, age at menarche; WC: ethnicity, educational attainment, marital status, annual family income, alcohol intake, age at first birth; CRP: ethnicity, educational attainment, marital status, annual family income, age at last period, years of hormone replacement therapy use; Glucose: marital status, annual family income, alcohol intake, age at last period, years of hormone replacement therapy use, age at first birth; Insulin: ethnicity, marital status, annual family income, smoking status, alcohol intake, years of hormone replacement therapy use, age at first birth; HOMA-IR: ethnicity, marital status, annual family income, smoking status, alcohol intake, age at first birth	High
Borgundvaag E., et al. 2017 [13]	Cohort (NHANES 2003–2004, 2005–2006)	5562 adults in the U.S. (48.4 ± 30 years)	ActiGraph 7164 (100 to 2019 CPM)	Quintile of LPA (ref. quintile 1)	Mortality (+): quintile 2; HR 0.72 (0.51, 1.03), quintile 3; HR 0.64 (0.42, 0.98), quintile 4; HR 0.75 (0.51, 1.11), quintile 5; HR 0.90 (0.62, 1.29)	Other physical activity intensity, age, sex, race/ethnicity, poverty-to-income ratio, education, smoking, alcohol, dietary fat, dietary saturated fat, dietary sodium, and accelerometer wear time	High
Howard B., et al. 2015 [14]	Cross-sectional (NHANES 2003–2004, 2005–2006)	4614 adults in the U.S. (46.8 ± 17 years) (2003 fasting sample, 851 OGTT sample)	ActiGraph 7164 (LLPA: 100 to 759 CPM, H LPA: 760 to 1951 CPM)	Every 1 SD increase in LLPA and HLPA	LLPA BMI (0): β − 0.31 (− 0.56, − 0.06), WC (+): β − 1.06 (− 1.65, − 0.47), SBP (0): RR 1.00 (1.00, 1.01), DBP (0): β 0.41 (− 0.42, 1.25), CRP (+): RR 0.91 (0.87, 0.95), HDL-C (0): RR 1.01 (1.00, 1.03), triglycerides (+) RR 0.96 (0.94, 0.98), LDL-C (0): β 0.01 (− 0.05, 0.07), plasma glucose (0): β 1.00 (1.00, 1.01), insulin (+):RR 0.92 (0.88, 0.96), HOMA β (+): RR 0.94 (0.92, 0.97), HOMA S (+): RR 1.08 (1.04, 1.12) and 2-h plasma glucose (0): RR 0.98 (0.95, 1.01) HLPA BMI (0): β 0.10 (− 0.14, 0.34), WC (0): β − 0.17 (− 0.73, 0.38, SBP (0): RR 1.00 (0.99, 1.00), DBP (0): β 0.39 (− 0.35, 1.14), CRP (+): RR 0.91 (0.86, 0.96), HDL-C (0): RR 1.01 (0.99, 1.02), triglycerides (+) RR 0.95 (0.92, 0.99), LDL-C (0): β 0.04 (− 0.03, 0.10), plasma glucose (0): β 1.00 (0.99, 1.00), insulin (+):RR 0.92 (0.87, 0.97), HOMA β (+): RR 0.98 (0.95, 1.00), HOMA S (+): RR 1.03 (1.00, 1.07) and 2-h plasma glucose (0): RR 0.97 (0.94, 1.00)	Age, sex, ethnicity, education, marital status, family poverty income ratio, smoking, alcohol intake, energy intake, saturated fat, medical characteristics, and WC, MET minutes of MVPA	Moderate
Schmid D., et al. 2016 [46]	Cohort (NHANES 2003–2004, 2005–2006)	3702 adults in the U.S. (50–80 years)	ActiGraph 7164 (100 to 2019 CPM)	Replacing 30 min of SB with an equal amount of LPA	All-cause mortality (+): HR 0.88 (0.84, 0.92), cardiovascular disease mortality (+): HR 0.88 (0.81, 0.95), cancer mortality (0): HR 0.93 (0.86, 1.01)	Age, sex, education, ethnicity, height, smoking, alcohol consumption, total dietary fat intake, total dietary fiber intake, mobility limitations, history of diabetes, history of coronary heart disease, history of congestive heart failure, history of stroke, history of cancer (where appropriate), total accelerometer wear time, and all activities except the one it was replaced for	Moderate
Hamer M., et al. 2014 [38]	Cross-sectional (Health Survey for England)	1947 adults in the U.K. (16–95 years)	ActiGraph GT1M (200 to 2019 CPM)	Tertiles of LPA (ref. the lowest tertile of LPA)	Psychological distress (+): OR 0.56 (0.37, 0.84) for the middle tertile, 0.73 (0.48, 1.12) for the highest tertile.	Age, sex, accelerometry wear time, smoking, alcohol, education, BMI, social occupational group employment long-term illness, and tertiles of MVPA	Moderate
Buman MP., et al. 2013 [25]	Cross-sectional (NHANES 2005–2006)	4130 adults in the U.S. (46.6 ± 18.5 years) (923 fasting samples)	ActiGraph 7164 (100 to 1951 CPM)	Replacing 30 min of SB with LPA	WC (0): RR 0.999 (0.996, 1.001), triglycerides (+): RR 0.981 (0.972, 0.991), insulin (+): RR 0.976, (0.962, 0.991), HDL-C (0): RR 1.003 (0.998, 1.008), HOMA β (+), HOMA S (+), SBP (−), BP (−), glucose (0), CRP (0), and LDL-C (0)	Age, sex, race/ethnicity, marital status, education, work status, poverty, smoking, depressive symptoms, energy intake, saturated fat, caffeine, alcohol use, general health rating, disease, sleep duration, and MVPA	Moderate
Fanning J., et al. 2016 [41]	Cross-sectional	247 adults in the U.S. (65.4 ± 4.6 years)	ActiGraph GT1M/ GT3X (51 to 1040 CPM)	Replacing 30 min of SB with LPA	Self-regulation (0), spatial working memory (0), and task switching (0)	Age, sex, race, total time, and other behavior.	Moderate

^a^+: beneficially associated/ favorable association, −: unfavorable association, 0: not associated

*SB* Sedentary behavior, *LPA* Light-intensity physical activity, *MVPA* Moderate-to-vigorous physical activity, *LLPA* Low light-intensity physical activity, *HLPA* High light-intensity physical activity, *MPA* Moderate physical activity, *VPA* Vigorous physical activity, *CPM* Counts per minute, *CRP* C-reactive protein, *WC* Waist circumference, *BMI* Body mass index, *LDL-C* Low density lipoprotein cholesterol, *HDL-C* High density lipoprotein cholesterol, *BP* Blood pressure, *DBP* Diastolic blood pressure, *SBP* Systolic blood pressure, *HOMA* Homeostasis model assessment, *OGTT* Oral glucose tolerance test, *MET* Metabolic equivalent, *SD* Standard deviation, *CI* Confidence interval, *OR* Odds ratio, *RR* Relative rate, *HR* Hazard ratio

### Definition of light-intensity physical activity

The majority of the studies assessed LPA using the ActiGraph accelerometer (Actigraph, LLC, FL) (*n* = 26, 84%); however, various cut off points were ued to determine LPA including 100–1951 counts per minute (CPM) [14, 15, 20–25], 100–2019 CPM [13, 26–30], 150–2689 CPM [31], 760–2019 CPM [32], 251–1951 CPM [33, 34], 100–1040 CPM [35], 100–759 CPM [36], 200–1998 CPM [37], 200–2019 CPM [38], 260–1952 CPM [39], 200–2689 CPM [40], and 51–1040 CPM [41]. Of the remaining four observational studies, two used the SenseWear Armband (BodyMedia, Inc., PA) with > 1.5 to < 3.0 Metabolic Equivalents (METs) [42, 43], one used the Actical (Mini Mitter Co., Inc., OR) with > 1.5 to < 3.0 METs [44], and one used the Active style Pro (Omron Healthcare Co., Ltd., Kyoto, Japan) with 1.6–2.9 METs [45]. One study [14] categorized LPA into two types according to intensity, namely, low light-intensity physical activity (LLPA) (100–759 CPM) and high light-intensity physical activity (HLPA) (760–1951 CPM).

### Health outcomes

The association between LPA and health outcomes reported in each study is presented in Table 1, and a summary of the included studies is listed in Table 2.

**Table 2:** Summary of studies analyzing associations of objectively measured light-intensity physical activity with health outcomes after adjustment for moderate-to-vigorous physical activity in adults

	Summary of all included studies	
	n/N	Association
Mortality	4/4 (100%)	Favorable (++)
Cardiometabolic risk factors		
WC	8/12 (67%)	Favorable (++)
Obese/ adiposity/ BMI	4/10 (40%)	Inconsistent (??)
Fat mass/ percent body fat	3/3 (100%)	Favorable (+)
SBP	1/6 (17%)	No association (00)
DBP	0/4 (0%)	No association (00)
High BP	1/5 (20%)	No association (00)
HDL-C	4/11(36%)	Inconsistent (??)
LDL -C	0/3 (0%)	No association (0)
Triglycerides	8/11 (73%)	Favorable (++)
Dyslipidemia	0/1 (0%)	No association (0)
Glucose	2/11 (18%)	No association (00)
Glycated hemoglobin	0/2 (0%)	No association (0)
Insulin/ diabetes	5/6 (83%)	Favorable (++)
HOMA β	1/2 (50%)	Inconsistent (?)
HOMA R	3/6 (50%)	Inconsistent (?)
CRP	1/4 (25%)	No association (0)
Metabolic syndrome	5/7 (71%)	Favorable (++)
Lipid accumulation	1/2 (50%)	Inconsistent (?)
Framingham risk score	0/1 (0%)	No association (0)
Mental health and cognitive function		
Mental health	1/1 (100%)	Favorable (+)
Well-being	1/1 (100%)	Favorable (+)
Cognitive health	1/2 (50%)	Inconsistent (?)
Other outcomes		
Medical multimorbidity	2/2 (100%)	Favorable (+)
Prostate-specific antigen	1/1 (100%)	Favorable (+)

No association (0): 0%–33% of the papers reported a statistically significant difference between LPA and outcomes, Indeterminate (?): 34–59% of the papers indicated a statistically significant difference, and favorable association (+)/ unfavorable association (−): 60%–100% of the papers showed a statistically significant favorable / unfavorable difference, respectively. When more than four studies supported an association or no association, it was coded as “00”, “++”, or “−−”. “??” indicates inconsistent findings

*BMI* Body mass index, *BP* Blood pressure, *DBP* Diastolic blood pressure, *SBP* Systolic blood pressure, *WC* Waist circumference, *HDL-C* High density lipoprotein cholesterol, *LDL-C* Low density lipoprotein cholesterol, *HOMA* Homeostasis model assessment, *CRP* C-reactive protein

N indicates the number of studies included in the review. n indicates the number of studies showing a favorable association

#### Cross-sectional studies

##### Cardiometabolic risk factors

LPA was found to have a favorable (++) association with waist circumference (WC) [eight [14, 23, 27, 29, 32, 35, 40, 45] of 12 studies (67%)], triglycerides [eight [14, 24, 25, 27, 31, 32, 37, 40] of 11 studies (73%)], insulin [five [14, 24, 25, 32, 35] of six studies (83%)], and presence of metabolic syndrome [five [27, 32, 35, 40, 45] of seven studies (67%)], whereas an inconsistent (??) association with BMI [four [23, 29, 35, 45] of 10 studies (40%)], and high density lipoprotein cholesterol (HDL-C) [four [21, 32, 40, 45] of 11 studies (36%)] was observed. There was no (00) association with systolic blood pressure (SBP) [one of six [14, 24, 25, 32, 42, 45] studies (16%)], diastolic blood pressure (DBP) [zero of four [14, 24, 42, 45] studies (0%)], high blood pressure (BP) [one of five [25, 27, 32, 40, 45] studies (20%)], and glucose [only two [14, 15] of 11 studies (18%)].

Evidence of the association with other cardiometabolic risk factors, such as fat mass, low density lipoprotein cholesterol (LDL-C), dyslipidemia, glycated hemoglobin, homeostasis model assessment (HOMA) β, HOMA R, C-reactive protein (CRP), lipid accumulation, and Framingham risk score were insufficient to determine the direction of association.

##### Mental health and cognitive function

One cross-sectional study reported that LPA was associated with a low risk of psychological distress [38]. Two studies on older adults analyzed the association between LPA and cognitive function; one study indicated that LPA was significantly associated with higher cognitive functioning [33] whereas the other study found no associations between LPA and spatial working memory and task-switching [41].

##### Other outcomes

Regarding the other health outcomes, there were two studies reporting favorable associations between LPA and the medical multimorbidity index [26, 29]. In addition, Loprinzi et al. found a favorable association between LPA and prostate-specific antigen concentrations [28].

#### Longitudinal studies

##### Mortality

Four cohort studies investigated the association between LPA and mortality [13, 30, 36, 46], and all studies reported a significant decrease in mortality risk. Matthews et al. [36] reported that those who performed 4 h/day of LPA had a 21% lower risk of mortality compared with those who did less LPA (3 h/day). Three studies (Fishman et al. [30], Matthews et al. [36], and Schmid et al. [46]) indicated that replacing sedentary time with LPA was associated with a lower risk of mortality; replacing 30 min of sedentary time with LPA was associated with a 20% reduction in mortality risk [30], replacing 60 min of sedentary time with LPA was associated with a lower hazard of death in the low-activity groups but not in the high-activity groups [36], and a 30 min increase in LPA concurrent with an equal decrease in sedentary time reduced mortality risk in both age groups (< 65 years and ≥ 65 years), respectively. The study by Borgundvaag et al. [13] analyzed the combined effects of LPA and MVPA and showed that modest to high LPA was associated with a significantly lower death rate than low LPA when MVPA was low in women.

##### Cardiometabolic risk factors

One cohort study for middle-aged Canadian adults reported that women in the highest tertiles of time performing LPA had lower fat mass, percent body fat, and central fat mass at 1 year follow-up, compared with women in the lowest and middle tertiles [44]. No significant effects were found in fat-free mass, peripheral fat mass, body weight, BMI, and WC [44].

##### Well-being

One cohort study for older adults in Taiwan showed that LPA was associated with three dimensions of well-being: psychological, learning and growth, and social well-being [22].

#### Duration of light-intensity physical activity bouts and health outcomes

Three studies [27, 28, 35] analyzed whether performing LPA in bouts of different length differently associated with health outcomes. Robson et al. [27] indicated that for every 30 min/day of activity, there was a significant 4% reduction in the relative odds of having metabolic syndrome for bouted (lasting at least 10 min) LPA, but not for sporadic (1–9 min) LPA. Bouted LPA was also associated with WC and triglyceride levels whereas sporadic LPA was not. On the other hand, Jefferis et al. [35] reported BMI, WC, fat mass index, and insulin as factors benefitting from sporadic LPA in older men. Another study by Loprinzi et al. [28] found that every 1-h increment of activity, there was a significant 18% reduction in the odds ratio of having elevated prostate-specific antigen concentration for 1-min bout of LPA, but not for ≥10-min bouts of LPA.

#### Intensity of light-intensity physical activity and health outcomes

One study [14] analyzed whether different light-intensity categories were associated with different cardiometabolic biomarkers. In the study by Howard et al. [14], both LLPA and HLPA were favorably associated with CRP, triglyceride levels, insulin, HOMA β, and HOMA R; and only LLPA showed significant favorable associations with WC. No association was observed in BMI, SBP, DBP, HDL-C, LDL-C, plasma glucose, and 2-h glucose.

#### Sex differences in associations between light-intensity physical activity and health outcomes

Three papers included in this review performed stratified analyses [13] or analyzed the interaction [14, 15] of sex differences in the effects of LPA on health outcomes. Borgundvaag et al. [13] found that a modest to high level of LPA was associated with a reduced mortality risk in women but not in men. In the study by Howard et al. [14], a significant interaction was observed by sex; association of HLPA with SBP tended to be beneficial in women only. On the other hand, Healy et al. [15] showed there was no sex interaction observed for the association between LPA and 2-h plasma glucose.

## Discussion

To the best of our knowledge, this is the first systematic review of epidemiological studies analyzing associations of objectively measured LPA with various health outcomes after adjustment for MVPA in adults and older adults. Our present systematic review shows that objectively measured LPA was inversely associated with all-cause mortality risk and was favorably associated with some cardiometabolic risk factors, including WC, triglyceride levels, insulin, and the presence of metabolic syndrome. Associations of LPA with BMI and HDL-C were inconsistent. On the other hand, there was no association with BP and glucose levels.

There is accumulating evidence regarding the health benefits of LPA, but further research is still needed. Approximately 90% of the studies included in our present review targeted the Western population, and thus the generalization of our findings to other populations should be performed with caution. Further studies on various populations should be carried out in the future. Furthermore, most of the studies were cross-sectional, and therefore more longitudinal research should be required to establish causality between LPA and health outcomes. Because only a small number of studies stratified the participants by age and sex, it was difficult to make any conclusions regarding the effects of age and sex. With increasing age, PA patterns change [47–49]; LPA would play a more important role in determining overall PA in the older adult population than in the adult population. Regarding sex, few studies performed stratified analyses or analyzed interactions to observe sex differences in the effects of LPA on health outcomes. Considering sex differences in metabolism (e.g., fat metabolism) and the fact that a number of previous studies using objective measurements reported that women engaged in more LPA than men [3, 15, 50], sex-stratified data are also warranted in future research.

The effects of LPA may depend on health outcomes. In the current review, LPA appears to be associated with some metabolic factors, including WC, triglyceride levels, insulin, BMI, and HDL-C, but not with BP. The physiological mechanisms underlying the observed associations are speculative. However, all factors associated with LPA are relevant to metabolic syndrome/diabetes followed by fat accumulation [51]. The increased activity of lipoprotein lipase and hormone-sensitive lipase that regulates lipid metabolism following muscular contractions causes decomposition of triglycerides into free fatty acid (FFA) [52–54], which reduces the triglycerides from the circulation. FFA is the primary fuel during low intensity activity [55]. Increased energy expenditure by LPA may be the reason for these the associations of LPA with fat-related metabolism. It is estimated that 1 h per day shift from SB to LPA (additional one MET-hour) would increase energy expenditure by about 3%, which could be significant to receive health benefits [25, 36]. On the other hand, LPA appears not to be associated with BP. Higher intensity PA may be needed for favorable effects on BP [56].

## Limitations

Several limitations should be considered when interpreting our present findings. First, this review used the P-counting method to summarize the research findings. It was not possible to perform a meta-analysis since there were some discrepancies and inconsistencies in how LPA was defined and analyzed between studies and populations. Analyzing accelerometer data using standardized methods will be helpful towards improving the quality of the scientific literature on PA and for maximizing comparability and synthesizing the results. Second, we cannot deny the effects of the differences in confounding adjustments that affect the associations between LPA and health outcomes. Third, our search strategy was restricted to studies written in English or Japanese, which might have resulted in language bias. Fourth, all of the studies included except one study [24] use a non-compositional approach. Therefore, the co-dependence of time-use domains is not totally taken into account. Further studies are needed to investigate combined effects of time spent in PA, SB and sleep on health markers using a compositional data analysis. Finally, there were no intervention studies and most of the studies were cross-sectional, which does not enable us to address the direction of causality. Therefore, additional longitudinal or intervention studies are needed to confirm these findings reported in this review. In the future, LPA should be measured and reported when intervention studies are performed, even on other time-use domains.

## Conclusions

This review highlights previous studies on the associations of objectively measured LPA and important health outcomes in adults. After adjustment for MVPA, LPA was inversely associated with all-cause mortality risk and was favorably associated with some cardiometabolic risk factors including WC, triglyceride levels, insulin, and the presence of metabolic syndrome. Although current global PA guidelines recommend only MVPA, promoting LPA may confer additional health benefits. Therefore, the inclusion of LPA in the PA recommendations should be considered in the future.

## Additional file

Additional file 1: Search Terms. (DOCX 27 kb)

## Abbreviations

BMI: Body mass index
BP: Blood pressure
CPM: Counts per minute
CRP: C-reactive protein
DBP: Diastolic blood pressure
HDL-C: High density lipoprotein cholesterol
HLPA: High light-intensity physical activity
HOMA: Homeostasis model assessment
LDL-C: Low density lipoprotein cholesterol
LLPA: Low light-intensity physical activity
LPA: Light-intensity physical activity
METs: Metabolic equivalents
MVPA: Moderate-to-vigorous physical activity
PA: Physical activity
SB: Sedentary behavior
SBP: Systolic blood pressure
WC: Waist circumference
