# Supplementary material for: Is objectively measured light-intensity physical activity associated with health outcomes after adjustment for moderate-to-vigorous physical activity in adults? A systematic review
Source: Int J Behav Nutr Phys Act. 2018 Jul 9;15:65. doi: 10.1186/s12966-018-0695-z (PMC6038338; doi:10.1186/s12966-018-0695-z)
Supplement: Supplementary file 1 — Search Terms. (DOCX 27 kb) [file 12966_2018_695_MOESM1_ESM.docx]

**Search terms**

(light intensity physical activit* OR low intensity physical activit* OR light intensity walking OR light intensity lifestyle OR light intensity exercise OR low intensity exercise OR light intensity activit* OR low intensity activit* OR light-intensity physical activit* OR low-intensity physical activit* OR light-intensity walking OR light-intensity lifestyle OR light-intensity exercise OR low-intensity exercise OR light-intensity activit* OR low-intensity activit* OR LPA OR LIPA) AND (actigraph OR objective* OR acceleromet* OR motion sensor OR CSA monitor OR activity monitor) AND (health OR mortality OR morbidity OR cardiovascular disease OR cancer OR biomarkers OR fasting plasma glucose OR glycosylated hemoglobin OR HbA1c OR triglycerides OR insulin OR total cholesterol OR LDL cholesterol OR HDL cholesterol OR CRP OR C-reactive protein OR metabolic syndrome OR body mass index OR BMI OR waist circumference OR hip circumference OR waist to hip ratio OR percent body fat OR lean body mass OR blood pressure OR mental disorders OR depressive disorders OR dementia OR cognition) AND (adult OR aged OR elderly OR older adults OR male OR female OR men OR women)
